# The prognostic nutritional index predicts all-cause mortality in critically ill patients with acute myocardial infarction

**DOI:** 10.1186/s12872-023-03350-4

**Published:** 2023-07-04

**Authors:** Yuekang Huang, Qunhui Zhang, Pengfei Li, Meixiang Chen, Ruixin Wang, Jiaman Hu, Jianing Chi, Hua Cai, Ningxia Wu, Lin Xu

**Affiliations:** 1 Department of Geriatric Cardiology, General Hospital of the Southern Theatre Command, Guangzhou, 510000 China; 2 The First School of Clinical Medicine, Southern Medical University, Guangzhou, 510000 China; 3 Branch of National Clinical Research Center for Geriatric Diseases, Chinese PLA General Hospital, Guangzhou, 510000 China; 4 Department of Cardiology, The First Affiliated Hospital, University of South China, Hengyang, 421001 China

**Keywords:** Prognostic nutritional index, Acute myocardial infarction, All-cause mortality, Prognosis, Intensive care unit

## Abstract

**Background:**

Malnutrition is common in patients with acute myocardial infarction (AMI) and is associated with a poor prognosis. The prognostic value of the prognostic nutritional index (PNI) in patients with AMI remains controversial. We aimed to explore the relationship between PNI and all-cause mortality in critically ill patients with AMI and evaluate the incremental prognostic value of PNI to commonly used prognostic assessment tools.

**Methods:**

The Medical Information Mart for Intensive Care-IV (MIMIC-IV) database was used to conduct a retrospective cohort analysis on 1180 critically ill patients with AMI. The primary endpoints were defined as 6-month and 1-year all-cause mortality. Cox regression analysis was used to investigate the relationship between admission PNI and all-cause mortality. The effect of adding PNI to sequential organ failure assessment (SOFA) score, or charlson comorbidity index (CCI) on its discriminative ability was assessed using C-statistic, net reclassification improvement (NRI), and integrated discrimination improvement (IDI).

**Results:**

Multivariate cox regression analysis demonstrated that the low PNI was regarded as an independent predictor of 1-year all-cause mortality in AMI patients admitted to ICU (adjusted Hazard Ratio: 95% CI = 1.75 (1.22–2.49)). The ROC test showed that admission PNI had a moderate predictive ability to predict all-cause mortality of critically ill patients with AMI. Furthermore, the net reclassification and integrated discrimination of the CCI alone model improved significantly with PNI. [C-statistic increased from 0.669 to 0.752, *p* < 0.001; NRI = 0.698, *p* < 0.001; IDI = 0.073, *p* < 0.001]. When PNI was added to the SOFA score, the C-statistic significantly improved from 0.770 to 0.805 (*p* < 0.001), and the NRI and IDI were estimated at 0.573 (*p* < 0.001) and 0.041 (*p* < 0.001), respectively.

**Conclusion:**

PNI could be a novel predictor for identifying patients at high risk of 1-year all-cause mortality in critically ill patients with AMI. The addition of PNI to the SOFA score or CCI may be useful for very early risk stratification.

**Supplementary Information:**

The online version contains supplementary material available at 10.1186/s12872-023-03350-4.

## Introduction

Acute myocardial infarction (AMI), a disastrous disease closely associated with inflammation, has been a fatal cardiovascular disease worldwide [1]. With the development of drug thrombolysis and early revascularization, the mortality of AMI has been reduced [2]. However, AMI patients need to complete continuous follow-ups to evaluate and improve their prognosis. Recently, the Gensini score and the SYNTAX score were used to predict the prognosis of AMI in clinics, but they could not be widely applied due to their complexity [3–5]. It was estimated that more than 50% of malnourished critically ill AMI patients admitted to the intensive care unit (ICU) could have more comorbidities and the risk of organ dysfunction [6, 7]. However, there are still no quick, easy, and effective predictors to evaluate their risk and nutritional status. Thus, it is urgent to investigate rapid and effective novel biomarkers to assess the prognosis of AMI patients admitted to the ICU.

The prognostic nutritional index (PNI), proposed by Mullen and his colleagues, was used to evaluate the prognosis of patients undergoing gastrointestinal surgery in 1980 [8]. Through optimization, PNI was formed by the concentration of serum albumin and the count of total lymphocytes [9]. In a word, PNI = 10 × serum albumin (g/dl) + 0.005 × total lymphocyte count (mm^3^). PNI reflected the inflammatory immune response and the status of nutrition, which played a critical role in appraising the prognosis of patients with multiple diseases, including cancer, lymphoma, infectious diseases, postoperative complication, etc [5, 10].

Recently, PNI has also been used to evaluate the prognosis of cardiovascular disease, including heart failure, ST-segment elevated myocardial infarction (STEMI), non-ST segment elevation myocardial infarction (NSTEMI), etc [11–13]. Several studies have found that PNI was an independent predictor for all-cause mortality in patients with elderly NSTEMI and STEMI patients after PCI, and was strongly associated with acute kidney injury of AMI patients [14, 15]. Others held the opposite view because they found that PNI did not show any better predictability than GRACE scores and it was even inferior to serum albumin alone in assessing prognosis [16]. In addition, few studies had focused on the relationship between PNI and AMI in the ICU. Compared with ordinary ward staff, these patients had more critical condition and adverse prognoses. Meanwhile, such patients were generally in a state of inflammation, which may aggravate the influence of malnutrition and induces poor prognoses and serious complications [17]. Consequently, this study focused on the relationship between PNI and poor prognosis of critically ill patients with AMI.

## Materials and methods

### Data source

This study was a single-center retrospective observational study. And the data was generated from the Medical Information Mart for Intensive Care-IV (MIMIC-IV, version 1.0) database, a large publicly available database comprising health-related data from patients who were admitted to the critical care units of the Beth Israel Deaconess Medical Center in 2008–2019 [18]. Acquisition of this online database was authorized by the Institutional Review Boards (IRB, Boston, MA, United States) of the Massachusetts Institute of Technology (MIT, Cambridge, MA, United States). All personal information was removed based on protective privacy. What was more, author Chen completed “protecting human subjects” training, and “Data or Specimens Only Research” training (certification number: 10,636,683). And She also finished data extraction which was conducted using PostgreSQL tools (version 10.18).

### Inclusion and exclusion criteria

The inclusion criteria were as follows. Patients with AMI (≥ 18 years) and finished 1-year follow-up records were screened in the subsequent analysis. The exclusion criteria could be listed as follows: (1) patients admitted less than 24 h in the ICU; (2) patients with malignant tumors; (3) patients with a hematologic disease which included lymphoma, leukemia, aplastic anemia, myelodysplastic syndrome, multiple myeloma; (4) patients lack of the counts of lymphocytes, the concentration of albumin; (5) patients with incomplete information. If the patients were admitted to the ICU more than once, only the first ICU admission data of the first hospitalization were included [19].

### Data extraction

The extraction of information included demographics, biochemical parameters, medical treatments, outcomes, etc. Demographic data contained age, sex, systolic blood pressure (SBP), and diastolic blood pressure (DBP). Risk factors contained hypertension, atrial fibrillation (AF), congestive heart failure (CHF), chronic kidney disease (CKD), non-STEMI, and STEMI, and diabetes mellitus (DM). Biochemical parameters included hemoglobin (HB), bicarbonate, creatinine (CR), potassium, and prothrombin time (PT). The scoring system included sequential organ failure assessment (SOFA) score system and charlson comorbidity index (CCI), and the highest SOFA value was selected for the subsequent analysis. Medical treatments contained antiplatelet drugs, β-blockers, statins, and angiotensin-converting enzyme inhibitors (ACEI)/angiotensin receptor blockers (ARB). The information about the initial ICU was used for the subsequent analysis.

### Prognostic nutritional index

The formula of PNI was listed as follows: PNI = 10 × serum albumin (g/dl) + 0.005 × total lymphocyte count (mm^3^) [20, 21].

### Endpoints and outcomes

The primary endpoints of this study were defined as 6-month and 1-year all-cause mortality. Additionally, other clinical outcomes focused on the in-hospital period were collected. These outcomes were regarded as ICU length of stay (LOS) and in-hospital all-cause mortality.

### Statistical analysis

All analysis was performed using R Studio software (Version 2022.02.1, Build 461, Boston, MA) and SPSS software (Version 22.0, IBM, US). All data were presented as mean ± standard deviation (SD) or a median with interquartile range (IQR). Firstly, the baseline characteristics were analyzed based on the occurrence of all-cause mortality during the follow-up and the cut-off value of PNI. The optimal cut-off value of PNI was obtained from the receiver operating curves of PNI, and the patients were divided into other 2 groups based on the cut-off value of PNI to investigate the function of PNI in the aspect of evaluating the prognosis of critically ill patients with AMI. Kolmogorov-Smirnov test was used to explore whether the variable conformed to a normal distribution [22]. Because continuous variables were not normally distributed, the Mann-Whitney U test was used to compare baseline characteristics. Categorical variables were analyzed and compared using the Chi-square test (χ^2^) and Fisher’s exact test. Mann-Whitney U test was carried out to compare the ICU LOS, and χ^2^ was used to analyze the time to occurrence of all-cause mortality in the two groups.

Spearman correlation analysis was used to examine the correlation between the PNI and all risk factors. The Kaplan-Meier method was performed to explore follow-up event rates. Time-event survival curves were drawn. The Log-Rank test was used to assess the difference. To determine whether the PNI was an independent predictor of all-cause mortality in AMI patients, univariate Cox regression analysis was used for each variable, and the relationship between the variable and the primary endpoints was obtained. Cumulative hazard functions and smoothed plots of the scaled Schoenfeld residuals were conducted to make the proportional hazards assumption [22]. Then variables were incorporated into multivariate cox proportional hazards regression models. And three models were constructed. Model 1 was adjusted for demographic data (gender and age) and PNI. Model 2 was a partially adjusted model, which contained variables with *p* < 0.05 in the previous univariate analysis and PNI. Model 3 was constructed based on Model 2, which included age, gender, initial ICU, SBP, DBP, hypertension, AF, CHF, CKD, STEMI, non-STEMI, DM, SOFA score system, CCI, HB, bicarbonate, CR, potassium, PT, antiplatelet drug, β-blockers, statins, and ACEI/ARB. In addition, the receiver operating characteristic curve (ROC) was performed to measure the sensitivity and specificity of admission PNI, SOFA score, and CCI.

Moreover, the area under the curve (AUC) was calculated to estimate the quality of admission PNI as a predictor of 6-month and 1-year all-cause mortality. Delong test was used to compare the difference in AUC between SOFA score, CCI, and PNI. Additionally, the time-dependent receiver operating curve (ROC) and area under the curve were performed to appraise the prognostic value and predictive efficacy of PNI. Finally, to evaluate whether an increased PNI had incremental predictive value for all-cause mortality, we compared baseline model composed of SOFA score or CCI with and without PNI. Net reclassification improvement (NRI) and integrated discrimination improvement (IDI) were obtained. A *p*-value of less than 0.05 was considered to be statistically significant.

## Results

### Baseline characteristics

**Fig. 1:**
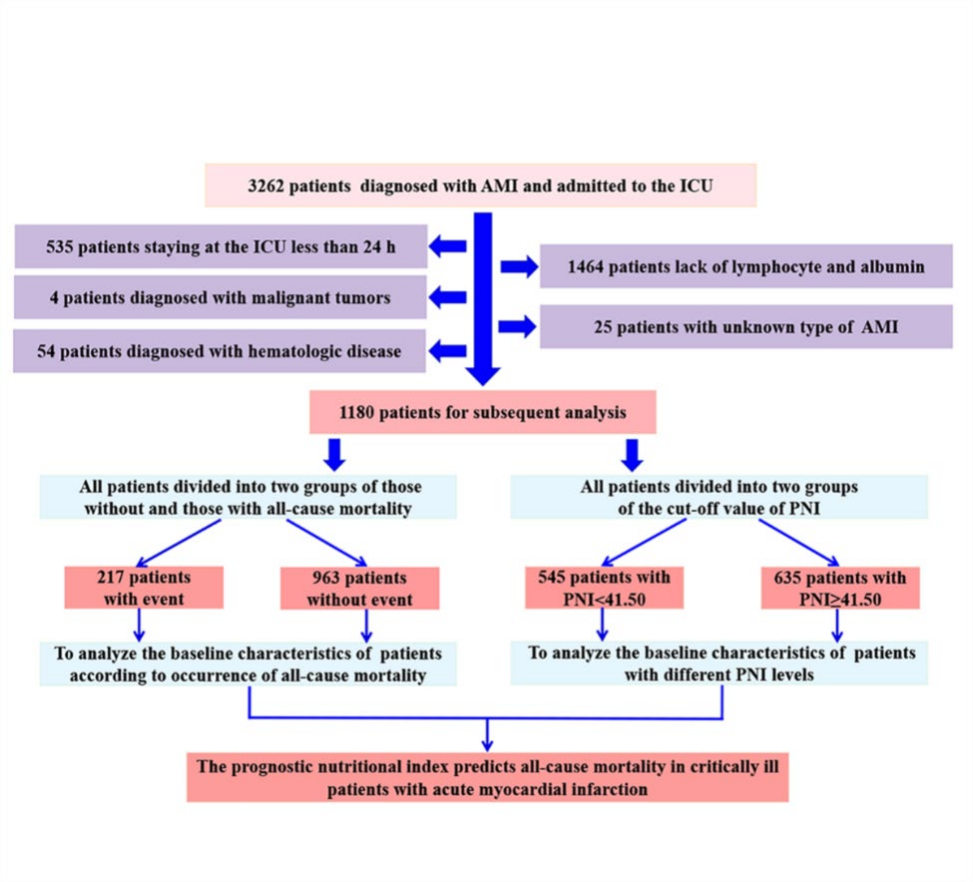
The flow chart of this study AMI: acute myocardial infarction; ICU: intensive care unit

A total of 3262 patients were firstly diagnosed with AMI when they were admitted to the ICU from the MIMIV-IV database. Of these patients, 2082 patients were excluded based on inclusion and exclusion criteria. 1180 patients were involved in the subsequent analysis (Fig. 1). Patients were divided into two groups those without and those with all-cause mortality. AMI patients who occurred death were indicated to be older, presented more complications (AF, CHF, and CKD), higher serum of CR, potassium, PT, SOFA score, CCI, and lower levels of PNI, SBP, HB, and bicarbonate (Table 1).

**Table 1:** Baseline characteristics of population according to the occurrence of all-cause death

Variables	Total (n = 1180)	With event (n = 217)	Without event (n = 963)	*p*-value
Demographics				
Age (years)	68.00 (59.00–76.00)	71.00 (62.00–79.00)	68.00 (59.00–75.00)	< 0.001
Male, n (%)	781 (66.19)	137 (63.13)	644 (66.87)	0.293
SBP (mmHg)	111.76 (104.97-119.78)	109.25 (101.90-117.23)	112.17 (105.50-120.21)	< 0.001
DBP (mmHg)	59.46 (53.78–66.36)	59.90 (53.66–67.36)	59.41 (53.83–66.18)	0.837
CCU, n (%)	804 (68.14)	89 (41.01)	715 (74.25)	< 0.001
**Risk factors, n (%)**				
Hypertension	396 (33.56)	38 (17.51)	358 (37.18)	< 0.001
AF	481 (40.76)	108 (49.77)	373 (38.73)	0.003
CHF	558 (47.29)	130 (59.91)	428 (44.44)	< 0.001
CKD	399 (33.81)	102 (47.00)	297 (30.84)	< 0.001
Non-STEMI	988 (83.73)	176 (81.11)	812 (84.32)	0.247
DM	543 (46.02)	106 (48.85)	437 (45.38)	0.354
**Biochemical parameters**				
HB (g/dL)	11.50 (9.60–13.40)	10.40 (9.00–12.00)	11.90 (9.90–13.60)	< 0.001
Bicarbonate (mmol/L)	23.00 (21.00–25.00)	22.00 (19.50–25.00)	23.00 (22.00–25.00)	< 0.001
CR (mg/dL)	1.30 (0.90–2.20)	2.20 (1.50–3.55)	1.10 (0.90–1.80)	< 0.001
Potassium (mmol/L)	4.60 (4.30–5.10)	4.80 (4.30–5.50)	4.60 (4.30-5.00)	< 0.001
PT (s)	15.20 (13.30-17.53)	15.90 (13.55-22.00)	15.00 (13.30–17.20)	< 0.001
**Cardiovascular medications, n (%)**				
Antiplatelet drugs	1087 (92.12)	177 (81.57)	910 (94.50)	< 0.001
β-blockers	986 (83.56)	130 (59.91)	856 (88.89)	< 0.001
ACEI/ARB	485 (41.10)	40 (18.43)	445 (46.21)	< 0.001
Statins	1061 (89.92)	166 (76.50)	895 (92.94)	< 0.001
**Score system**				
SOFA score	7.00 (5.00–10.00)	11.00 (8.00–13.00)	6.00 (4.00–9.00)	< 0.001
CCI	7.00 (5.00–9.00)	9.00 (7.00–10.00)	7.00 (5.00–9.00)	< 0.001
**PNI**	42.25 (35.51–48.55)	34.80 (29.52–41.05)	43.70 (37.70-49.55)	< 0.001

A median with IQR: n (%). SBP: systolic blood pressure; DBP: diastolic blood pressure; CCU: cardiac care unit; AF: atrial fibrillation; CHF: congestive heart failure; CKD: chronic kidney disease; non-STEMI: non-ST segment elevation myocardial infarction; DM: diabetes mellitus; HB: hemoglobin; CR: creatinine; PT: prothrombin time; ACEI/ARB: angiotensin-converting enzyme inhibitors/angiotensin receptor blockers; SOFA: sequential organ failure assessment; CCI: Charlson comorbidity index; PNI: prognostic nutritional index. A *p* value < 0.05 was regarded as statistical significance

To analyze the baseline situation of AMI patients with different PNI levels and identify the relationship between PNI and all-cause mortality in AMI patients, ROCs were used to obtain optimal cut-off values [23]. According to the cut-off value, these patients were divided into the high PNI group and the low PNI group, which contributed to further analysis of the relationship between PNI and endpoints. The results showed that the superior cut-off of PNI for predicting 6-month and 1-year all-cause mortality was 41.50 (Figure S1, A, B). As presented in Fig. 1; Table 2, AMI patients were divided into two groups based on the cut-off value of PNI (group 1: n = 545, PNI < 41.50; group 2: n = 635, PNI ≥ 41.50). There were remarkable differences between the two groups in terms of age, male, first care unit, hypertension, AF, CHF, CKD, HB, bicarbonate, CR, PT, cardiovascular medications, ICU LOS, in-hospital, six-months mortality, and 1-year mortality (Table 2). Compared to the group with high level of PNI, the patients with low PNI had a longer ICU LOS and a greater risk of in-hospital mortality.

**Table 2:** Baseline characteristics of the population according to the cut-off value of PNI

Variables	Group 1 (n = 545)	Group 2 (n = 635)	*p*-value
Demographics			
Age (years)	70 (61.00–78.00)	67.00 (58.00–74.00)	< 0.001
Male, n (%)	336 (61.65)	445 (70.08)	0.002
SBP (mmHg)	110.97 (103.67-120.55)	112.13 (106.30-119.31)	0.061
DBP (mmHg)	59.30 (54.07–66.82)	59.53 (53.64–65.54)	0.828
CCU, n (%)	254 (46.61)	550 (86.61)	< 0.001
**Risk factors, n (%)**			
Hypertension	124 (22.75)	272 (42.83)	< 0.001
AF	253 (46.42)	228 (35.91)	< 0.001
CHF	324 (59.45)	234 (36.85)	< 0.001
CKD	239 (43.85)	160 (25.20)	< 0.001
Non-STEMI	456 (83.67)	532 (83.78)	0.959
DM	246 (45.14)	297 (46.77)	0.574
**Biochemical parameters**			
HB (g/dL)	10.20 (8.70–11.90)	12.70 (11.10–14.10)	< 0.001
Bicarbonate (mmol/L)	22.00 (20.00–25.00)	24.00 (22.00–26.00)	< 0.001
CR (mg/dL)	1.70 (1.20–3.10)	1.10 (0.80–1.40)	< 0.001
Potassium (mmol/L)	4.70 (4.20–5.30)	4.60 (4.40-5.00)	0.912
PT (s)	15.30 (13.30-18.95)	15.10 (13.40–17.00)	0.028
**Cardiovascular medications, n (%)**			
Antiplatelet drugs	472 (86.61)	615 (96.85)	< 0.001
β-blockers	400 (73.39)	586 (92.28)	< 0.001
ACEI/ARB	174 (31.93)	311 (48.98)	< 0.001
Statins	454 (83.30)	607 (95.59)	< 0.001
**Score system**			
SOFA score	8.00 (6.00–12.00)	6.00 (4.00–8.00)	< 0.001
CCI	8.00 (6.00–10.00)	6.00 (5.00–8.00)	< 0.001
**Outcome**			
ICU LOS (days)	4.10 (2.36–7.37)	2.22 (1.35–3.85)	< 0.001
**All-cause-mortality, n (%)**			
In**-**hospital mortality	136 (24.95)	44 (6.93)	< 0.001
6-month mortality	160 (29.36)	50 (7.87)	< 0.001
1-year mortality	167 (30.64)	50 (7.87)	< 0.001

A median with IQR, n (%). SBP: systolic blood pressure; DBP: diastolic blood pressure; CCU: cardiac care unit; AF: atrial fibrillation; CHF: congestive heart failure; CKD: chronic kidney disease; non-STEMI: non-ST segment elevation myocardial infarction; DM: diabetes mellitus; HB: hemoglobin; CR: creatinine; PT: prothrombin time; ACEI/ARB: angiotensin-converting enzyme inhibitors/angiotensin receptor blockers; SOFA score: sequential organ failure assessment score; CCI: charlson comorbidity index; ICU LOS: intensive care unit length of stay. A *p* value < 0.05 was regarded as statistical significance. Group 1: PNI < 41.50, Group 2: PNI ≥ 41.50

### Correlation between the PNI and risk factors

To explore the association between underline parameters and PNI, spearman correlation analysis was conducted. As presented in Table 3, the PNI was positively correlated with HB and bicarbonate (*r* > 0, *p* < 0.05). However, it was negatively correlated with age, CR, SOFA score, and CCI (*r* < 0, *p* < 0.05). No important correlation was found between the PNI and SBP, DBP, potassium, and PT (Table 3).

**Table 3:** Correlations between baseline PNI and risk factors

Variables	PNI	
	r-value	*p*-value
Age (years)	-0.148	< 0.001
SBP (mmHg)	0.052	0.071
DBP (mmHg)	-0.022	0.450
HB (g/dL)	0.491	< 0.001
Bicarbonate (mmol/L)	0.202	< 0.001
CR (mg/dL)	-0.418	< 0.001
Potassium (mmol/L)	0.006	0.838
PT (s)	-0.047	0.107
SOFA score	-0.331	< 0.001
CCI	-0.327	< 0.001

PNI: prognostic nutritional index; SBP: systolic blood pressure; DBP: diastolic blood pressure; HB: hemoglobin; CR: creatinine; PT: prothrombin time; SOFA score: sequential organ failure assessment score; CCI: charlson comorbidity index; A *p* value < 0.05 was regarded as statistical significance.

### Admission PNI and endpoints

**Fig. 2:**
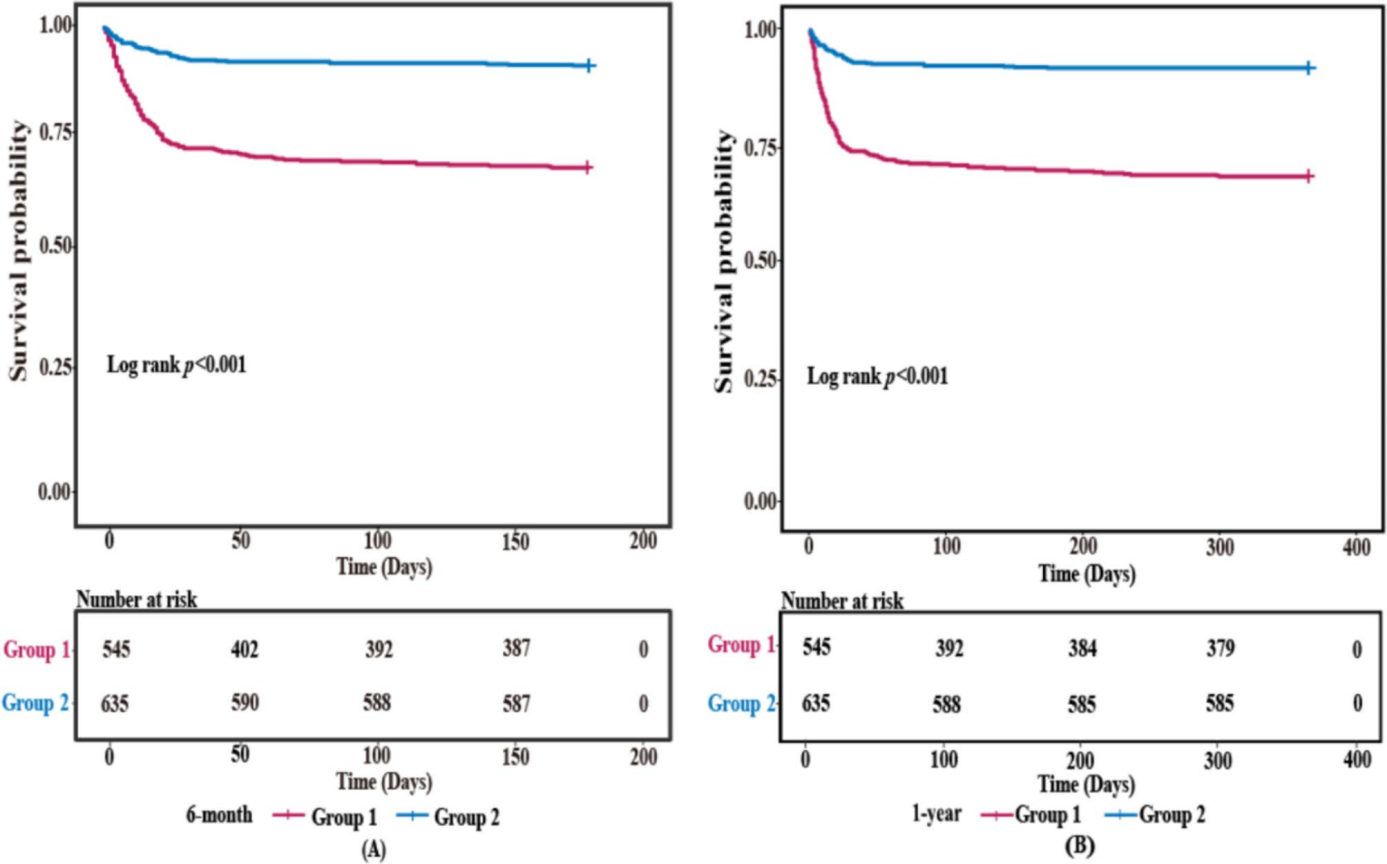
Kaplan-Meier survival curves for all-cause mortality across the PNI (**A**) Kaplan-Meier curve of six-month all-cause mortality. (**B**) Kaplan-Meier curve of one-year all-cause mortality

To investigate the endpoints of these patients, Kaplan-Meier analysis was conducted to present their relationships (Fig. 2). As depicted in Fig. 2, during six months and a year, the cumulative incidence of all-cause mortality of AMI patients in Group 1 was higher than it was in Group 2 (log-rank test, *p* < 0.001).

### Admission PNI as a predictor of the clinical endpoints

To investigate whether PNI was an independent predictor of all-cause mortality in AMI patients, a univariate cox regression analysis was performed to identify significant factors associated with six-month and 1-year all-cause mortality. As shown in Table 4, age, SBP, CCU, hypertension, AF, CHF, CKD, HB, bicarbonate, CR, potassium, PT, antiplatelet drugs, β-blockers, ACEI/ARB, Statins, SOFA score, CCI and PNI were identified as risk factors for six-month and 1-year all-cause mortality. Furthermore, compared to the high PNI group, the unadjusted HR (95% CI) for risk of all-cause mortality with per unit decrease in the PNI was 4.23 (95% Cl: 3.08–5.81, *p* < 0.001) for 6-month risk of all-cause mortality and 4.43 (95% Cl: 3.23–6.08, *p* < 0.001) for 1-year risk of all-cause mortality (Table 4). Multivariate Cox proportional hazards regression analysis demonstrated that the PNI, whether regarded as a continuous or categorical factor, still kept significant after adjusting for confounders. For per unit decrease in the PNI (categorical), the risk of incident six-month all-cause mortality increased by 82% (*p* = 0.001) and 62% (*p* = 0.003) in models 2 and 3, respectively (Table 5). Similarly, 1-year all-cause mortality increased by 93% (*p* < 0.001) and 75% (*p* = 0.002) in models 2 and 3, respectively (Table 5).

**Table 4:** Univariate Cox regression analyses for 6-month and 1-year all-cause mortality

Variables	6-month		1-year	
	HR (95%CI)	*p*-value	HR (95%CI)	*p*-value
Age	1.03 (1.01–1.04)	< 0.001	1.02 (1.01–1.03)	< 0.001
Male	0.84 (0.64–1.12)	0.230	0.85 (0.65–1.12)	0.256
SBP	0.98 (0.97–0.99)	0.001	0.98 (0.97–0.99)	0.001
DBP	1.00 (0.99–1.01)	0.992	1.00 (0.99–1.02)	0.890
CCU	0.29 (0.22–0.38)	< 0.001	0.28 (0.22–0.37)	< 0.001
Hypertension	0.38 (0.27–0.54)	< 0.001	0.39 (0.27–0.55)	< 0.001
DM	1.07 (0.82–1.40)	0.632	1.12 (0.86–1.47)	0.393
AF	1.52 (1.16-2.00)	0.002	1.48 (1.14–1.94)	0.004
CHF	1.70 (1.29–2.24)	< 0.001	1.73 (1.32–2.28)	< 0.001
CKD	1.82 (1.39–2.39)	< 0.001	1.82 (1.39–2.37)	< 0.001
Non-STEMI	0.75 (0.53–1.05)	0.097	0.78 (0.56–1.10)	0.153
HB	0.85 (0.81–0.90)	< 0.001	0.85 (0.80–0.90)	< 0.001
Bicarbonate	0.93 (0.89–0.97)	< 0.001	0.94 (0.90–0.97)	< 0.001
CR	1.16 (1.11–1.21)	< 0.001	1.17 (1.12–1.22)	< 0.001
Potassium	1.30 (1.12–1.50)	0.001	1.31 (1.14–1.52)	< 0.001
PT	1.03 (1.02–1.05)	< 0.001	1.03 (1.02–1.05)	< 0.001
Antiplatelet drugs	0.30 (0.21–0.42)	< 0.001	0.31 (0.22–0.43)	< 0.001
Beta-blockers	0.21 (0.16–0.28)	< 0.001	0.22 (0.17–0.29)	< 0.001
ACEI/ARB	0.26 (0.18–0.37)	< 0.001	0.29 (0.20–0.40)	< 0.001
Stains	0.27 (0.20–0.37)	< 0.001	0.28 (0.21–0.39)	< 0.001
SOFA score	1.23 (1.19–1.27)	< 0.001	1.22 (1.19–1.26)	< 0.001
CCI	1.17 (1.12–1.23)	< 0.001	1.18 (1.13–1.23)	< 0.001
PNI (continuous)	0.93 (0.91–0.94)	< 0.001	0.93 (0.91–0.94)	< 0.001
PNI (categorical)		< 0.001		< 0.001
Group 1	4.23 (3.08–5.81)	< 0.001	4.43 (3.23–6.08)	< 0.001
Group 2	Reference		Reference	

SBP: systolic blood pressure; DBP: diastolic blood pressure; CCU: cardiac care unit; DM: diabetes mellitus; AF: atrial fibrillation; CHF: congestive heart failure; CKD: chronic kidney disease; non-STEMI: non-ST segment elevation myocardial infarction; HB hemoglobin; CR: creatinine; PT: prothrombin time; ACEI/ARB: angiotensin-converting enzyme inhibitors/angiotensin receptor blockers; SOFA score: sequential organ failure assessment score; CCI: charlson comorbidity index; PNI: prognostic nutritional index; HR: hazard ratio; CI: confidence interval. A *p* value < 0.05 was regarded as statistical significance. Group 1: PNI < 41.50, Group 2: PNI ≥ 41.50

**Table 5:** Multivariate Cox regression analyses for 6-month and 1-year all-cause mortality

PNI	HR (95%Cl)		
	Model 1	Model 2	Model 3
6-month			
Per 1 unit increase	0.93 (0.91–0.94)**	0.97 (0.95–0.99)**	0.97 (0.95–0.99)*
Group 1 is used as reference			
Group 1	1 (Reference)	1 (Reference)	1 (Reference)
Group 2	0.25 (0.18–0.34)**	0.55 (0.39–0.78)*	0.62 (0.43–0.88)*
Group 2 is used as reference			
Group 1	4.01 (2.91–5.53)**	1.82 (1.29–2.59)*	1.62 (1.14–2.32)*
Group 2	1 (Reference)	1 (Reference)	1 (Reference)
**1-year**			
Per 1 unit increase	0.92 (0.91–0.94)**	0.97 (0.95–0.98)**	0.97 (0.95–0.99)*
Group 1 is used as reference			
Group 1	1 (Reference)	1 (Reference)	1 (Reference)
Group 2	0.24 (0.17–0.32)**	0.52 (0.37–0.73)**	0.57 (0.40–0.82)*
Group 2 is used as reference			
Group 1	4.25 (3.09–5.84)**	1.93 (1.37–2.74)**	1.75 (1.22–2.49)*
Group 2	1 (Reference)	1 (Reference)	1 (Reference)

Model 1: adjusted for Age and Gender

Model 2: adjusted for variables with *p*-value < 0.05 in univariate analysis, including Age, SBP, CCU, Hypertension, AF, CHF, CKD, HB, Bicarbonate, CR, Potassium, PT, Antiplatelet drugs,Beta-blockers, ACEI/ARB, Statins, SOFA score, CCI.

Model 3: adjusted for Age, Gender, SBP, DBP, CCU, Hypertension, DM, AF, CHF, CKD, non-NSTEMI, HB, bicarbonate, CR, Potassium, PT, Antiplatelet drugs, Beta-blockers, ACEI/ARB, Statins, SOFA score, CCI.

PNI: prognostic nutritional index; SBP: systolic blood pressure; DBP: diastolic blood pressure; CCU: cardiac Care Unit; DM: diabetes mellitus; AF: atrial fibrillation; CHF: congestive heart failure; CKD: chronic kidney disease; non-STEMI: non ST segment elevation myocardial infarction; HB: hemoglobin; CR: creatinine; PT: prothrombin time; ACEI/ARB: angiotension converting enzyme inhibitors/angiotensin receptor blockers; SOFA score: sequential organ failure assessment score; CCI: charlson comorbidity index; HR: hazard ratio; CI: confidence interval. Note: *p < 0.05, **p < 0.001

### The dignostic efficacy of PNI, CCI and SOFA score

**Fig. 3:**
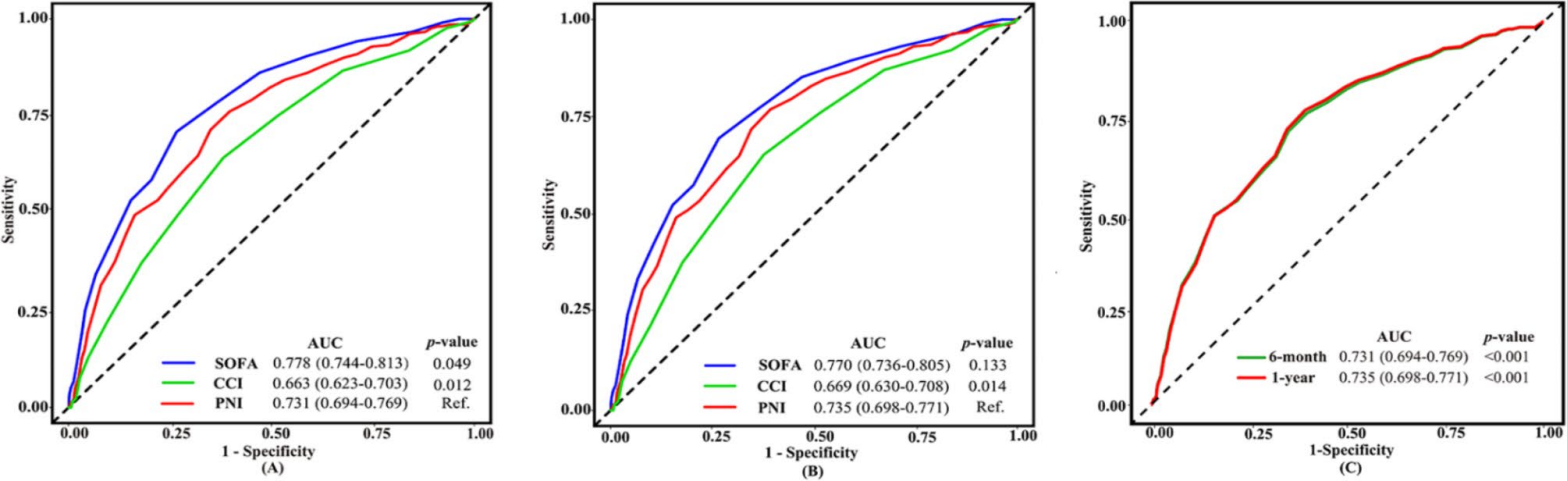
ROC curves for the prediction of 6-month and 1-year all-cause mortality (**A**) About 6-month. (**B**) About 1-year. (**C**) Time-dependent ROC curves between the PNI and all-cause mortality SOFA: sequential organ failure assessment score; CCI: charlson comorbidity index; HR: hazard ratio; PNI: prognostic nutritional index; AUC: the area under the curve

To clarify the prognostic efficacy of PNI, CCI, and SOFA score for critically ill AMI, ROC and AUC were carried out. These results showed that the AUC of admission PNI, CCI, and SOFA score in predicting 6-month all-cause mortality were 0.731 (95% CI: 0.694–0.769, *p* < 0.001), 0.663 (95% CI: 0.623–0.703, *p* < 0.001) and 0.778 (95% CI: 0.744–0.813, *p* < 0.001), respectively. The AUC of admission PNI, CCI, and SOFA score in predicting 1-year all-cause mortality were 0.735 (95% CI: 0.698–0.771, *p* < 0.001), 0.669 (95% CI: 0.630–0.708, *p* < 0.001), and 0.770 (95% CI: 0.736–0.805, *p* < 0.001), respectively. The diagnostic efficacy of PNI was better than CCI in predicting all-cause mortality at 6-month and 1-year (*p* = 0.012, *p* = 0.014, respectively). The diagnostic efficacy of SOFA scores was superior to PNI (*p* = 0.049) in 6-month all-cause mortality. However, there was no difference between the SOFA score and PNI in predicting all-cause mortality at 1-year (*p* = 0.133). (Fig. 3, A, B).

To further evaluate the diagnostic value of PNI, a time-dependent ROC analysis was performed. And the results showed that AUC of 0.731 (95%CI: 0.694–0.769, *p* < 0.001) at 6 months, and 0.735 (95%CI: 0.698–0.771, *p* < 0.001) at 1 year (Fig. 3, C). Thus, PNI was relatively stable in predicting all-cause mortality in critically ill AMI patients within 1 year.

### Risk discriminative power of PNI

To explore whether PNI could enhance the predictive efficacy of traditional prognostic assessment tools in critically ill patients, the PNI (continuous variable) was added to the traditional prognostic assessment tools (SOFA score and CCI) and constructed new models. Adding the PNI to a baseline model with SOFA score improved the prediction of 1-year all-cause mortality (*p* < 0.001, Table 6). Reclassification of patients was performed using the NRI and IDI. The NRI and IDI for 1-year all-cause mortality were significantly increased after adding the PNI to a baseline model with SOFA score (all *p* < 0.001, Table 6). Similarly, compared to the baseline model with CCI, the C-statistic of the new model integrated with CCI and PNI was higher (new model vs. base model: 0.752 (0.726–0.776) vs. 0.669 (0.630–0.708), *p* < 0.001). According to the NRI and IDI, we find that the addition of PNI to CCI significantly improved the risk reclassification for the risk of 1-year all-cause mortality (NRI: 0.698, *p* < 0.001; IDI: 0.073, *p* < 0.001).

**Table 6:** Discrimination and reclassification for 1-year all-cause mortality with the addition of PNI to traditional prognostic assessment tools

	C-statistic (95%CI)	*p*-value	Continuous NRI (95%CI)	*p*-value	IDI (95%CI)	*p*-value
**SOFA**	0.770 (0.736–0.805)	Ref		Ref		Ref
**SOFA + PNI**	0.805 (0.781–0.827)	< 0.001	0.573 (0.432–0.713)	< 0.001	0.041 (0.026–0.057)	< 0.001
**CCI**	0.669 (0.630–0.708)	Ref		Ref		Ref
**CCI + PNI**	0.752 (0.726–0.776)	< 0.001	0.698 (0.560–0.836)	< 0.001	0.073 (0.055–0.091)	< 0.001

AUC: the area under the curve; NRI: net reclassification improvement; IDI: integrated discrimination improvement; SOFA: sequential organ failure assessment score; CCI: charlson comorbidity index; PNI: prognostic nutritional index

## Discussion

AMI is one of the most acute and critical cardiovascular diseases worldwide [1]. Therefore, early prognosis assessment of these patients is very significant. However, the commonly used prognostic indicators are still insufficient. Declining hemoglobin content, as a novel biomarker, can be regarded as an effective mortality predictor for patients with AMI, which provides a promising perspective to evaluate the prognosis of patients with AMI [24]. In addition, malnutrition is a common phenomenon in patients with AMI and is strongly associated with increased mortality and cardiovascular events [6, 25, 26]. What is more, it is known to us all that inflammatory response has been proven to play a significant role in the whole process of AMI [27]. For critically ill patients, the interaction of malnutrition and inflammation may exacerbate the occurrence of poor outcomes including clinical complications, longer hospital stays, and major cardiovascular events (MACEs) [28–30]. Many assessment tools are used to evaluate nutritional status. NRS-2002 and NUTRIC are used in many situations. However, they are not universally accepted scoring systems for critically ill patients [31]. The NRS-2002 consists of BMI, percentage of recent weight loss, and recent changes in food intake [32]. The NUTRIC score is composed of age, acute physiology and chronic health evaluation, SOFA score, number of comorbidities, days from hospital admission to ICU admission, and serum interleukin-6 [33]. The European Society of Parenteral and Enteral Nutrition (ESPEN) and the American Society for Parenteral and Enteral Nutrition/Society for Critical Care Medicine have rejected to recommend among the two scores [34]. ESPEN recommends against these scores. At present, there are few studies about the nutritional status of critically ill AMI patients admitted to ICU. Existing methods for assessing the prognosis of critically ill patients with AMI are complicated and difficult to implement, which limits their clinical application [5]. Thus, it is necessary for us to screen novel biomarkers to evaluate the prognosis of AMI.

In this study, the primary outcomes are as follows: (1) Adjusting for potential risk factors, lower PNI was associated with an increased risk of all-cause mortality in AMI patients admitted to the ICU. (2) In the aspects of PNI predicting all-cause mortality in AMI patients, outcomes demonstrated PNI was superior to CCI in evaluating all-cause mortality in critically ill AMI patients. There was no significant difference between the AUC of PNI and SOFA score at 1-year all-cause mortality. Therefore, PNI was a convincing predictor of 6-month and 1-year all-cause mortality. (3) Adding the PNI to the traditional prognosis assessment tools could improve outcome prediction in critically ill patients with acute myocardial infarction.

PNI was calculated based on serum albumin concentration and total lymphocyte count in peripheral blood, which was mainly used for evaluating the immune and nutritional status of patients [20]. Serum albumin, as one component of PNI, was an important extracellular antioxidant. When serum albumin stayed at normal concentrations, it played an important role in inhibiting platelet activation and aggregation and vascular endothelial cell apoptosis [35]. Plakht et al. retrospectively recruited patients who were admitted to a tertiary medical center for AMI and discharged alive and demonstrated that serum albumin was associated with all-cause mortality of AMI [36]. Other studies have found that low levels of serum albumin on admission were also independent predictors of long-term all-cause, cardiovascular, and cardiac death in patients with AMI [37]. Lymphocytes, another component of PNI, are a kind of immune cells in the body and closely related to the progression of inflammation and have been proven to be involved in coordinating the complex and dynamic inflammatory response of AMI [38]. Studies have indicated that the reduction of the count of regulatory T cells was associated with an increase in myocardial infarction [39]. Increased lymphocyte count could reflect a moderate immune response, and a stable and static inflammatory state [12]. Arbel et al. found that lymphopenia was independently associated with the occurrence of complications and death after AMI [40]. Thus, serum albumin and lymphocytes did play an important role in the process of AMI. Recently, PNI has also been used to assess the prognosis and complications of various coronary artery disease (CAD), including acute heart failure [11], dilated cardiomyopathy [41], stable CAD [42], etc. Raposeiras et al. followed up 5062 acute coronary syndrome patients and indicated the role of PNI in predicting ACS all-cause mortality and MACEs [6]. Previous studies have demonstrated the relationship between the PNI and acute heart failure, STEMI, NSTEMI and renal insufficiency. More importantly, the results of our study have shown that PNI could be an effective predictor of all-cause mortality in critically ill patients with AMI. Consequently, PNI was a significant predictor for CAD.

The CCI, recognized as the gold standard for evaluating comorbidity, was the most commonly used comorbidity index in clinical and was proven to predict long-term mortality in different clinical populations, including medical, surgical, and ICU [43]. High CCI was associated with adverse events. The distinguished performance of CCI was inferior to other prognostic indices for ICU or trauma patients [44]. The components of PNI included albumin and lymphocytes, which could reflect the immune inflammation and nutritional status of the body. In this study, we found that the AUC of PNI was larger than that of CCI no matter in 6-month or 1-year all-cause mortality (*p* < 0.05). Compared with CCI, PNI could be more suitable for patients with critical diseases accompanied by a high pro-inflammatory state.

SOFA scores were made up of six assessment scores for different organs or systems, including respiration, blood clotting, liver, circulation, nerve, and kidneys. So it was often used to describe and quantify the risk and severity of organ failure in critically ill patients. The maximum value of the total SOFA score represented cumulative organ dysfunction [45]. Patients in the ICU suffered from a higher incidence of organ dysfunction related to higher mortality and poor prognosis [7]. Ferreira et al. showed that SOFA score was a good indicator of poor prognosis of critically ill patients in the ICU. Other studies have shown that SOFA score provided potentially valuable prognostic information on clinical outcomes when applied to patients with AMI. [46]. In our study, we found that there was no statistical significance between the AUC of PNI and that of SOFA score in 1-year all-cause mortality. These results indicated that PNI was a reliable predictor of 1-year all-cause mortality. Compared to SOFA score, PNI was a simple, data-accessible predictor that could be used to rapidly evaluate the prognosis of patients.

In our study, the results suggest that the PNI level at admission is likely to accurately and efficiently enhance the predictive value of SOFA score or CCI for adverse cardiovascular events in critically ill patients with AMI. We found that PNI should be considered when using SOFA score or CCI to assess the prognosis of critically ill patients with AMI, which could provide clinicians with a more comprehensive assessment of the prognosis of these patients.

### Limitation

This study was a single-center retrospective study, and some important information may be omitted such as smoking, drinking history and death reason. Patients’ information was identified using ICD-9, ICD-10 and unique ID from the MIMIC-IV database rather than clinical diagnostic criteria, so some information was not specific. Only the PNI measured for the first time after admission was selected for this study. Random errors might occur. Changes in PNI level at different periods might provide additional prognostic information. Despite our convincing results, larger clinical studies should be designed for validation.

## Conclusion

Our study suggests that lower admission PNI was independently associated with 6-month and 1-year all-cause mortality in critically ill patients with AMI. Patients with low PNI are faced with a significant mortality risk and have a longer ICU LOS. PNI may be a simple clinical maker to predict risk stratification in AMI patients admitted to ICU. Moreover, PNI could enhance the predictability of the model with SOFA score or CCI alone for the prognosis of critically ill patients with AMI.

## Electronic supplementary material

Below is the link to the electronic supplementary material.

Additional File 1:

## Data Availability

The original data of our study are available from the corresponding author upon reasonable request.

## Abbreviations

AMI: Acute myocardial infarction
PNI: Prognostic nutritional index
MIMIC-IV: Medical Information Mart for Intensive Care-IV
ROC: Receiver operating characteristic curve
AUC: Area under the receiver operating characteristic curve
SOFA: Sequential organ failure assessment
CCI: Charlson comorbidity index
NRI: Net reclassification improvement
IDI: Integrated discrimination improvement
HR: Hazard ratio
CI: Confidence interval
ICU: Intensive care unit
STEMI: ST-segment elevated myocardial infarction
NSTEMI: Non-ST segment elevation myocardial infarction
SBP: Systolic blood pressure
DBP: Diastolic blood pressure
AF: Atrial fibrillation
CHF: Congestive heart failure
CKD: Chronic kidney disease
DM: Diabetes mellitus
HB: Hemoglobin
CR: Creatinine
PT: Prothrombin time
ACEI: Angiotensin-converting enzyme inhibitors
ARB: Angiotensin receptor blockers
LOS: Length of stay
SD: Standard deviation
IQR: Interquartile range
MACEs: Major cardiovascular events
ESPEN: The European Society of Parenteral and Enteral Nutrition.
