# Supplementary material for: The prognostic nutritional index predicts all-cause mortality in critically ill patients with acute myocardial infarction
Source: BMC Cardiovasc Disord. 2023 Jul 4;23:339. doi: 10.1186/s12872-023-03350-4 (PMC10318819; doi:10.1186/s12872-023-03350-4)
Supplement: Supplementary file 1 — Additional File 1: [file 12872_2023_3350_MOESM1_ESM.pdf]

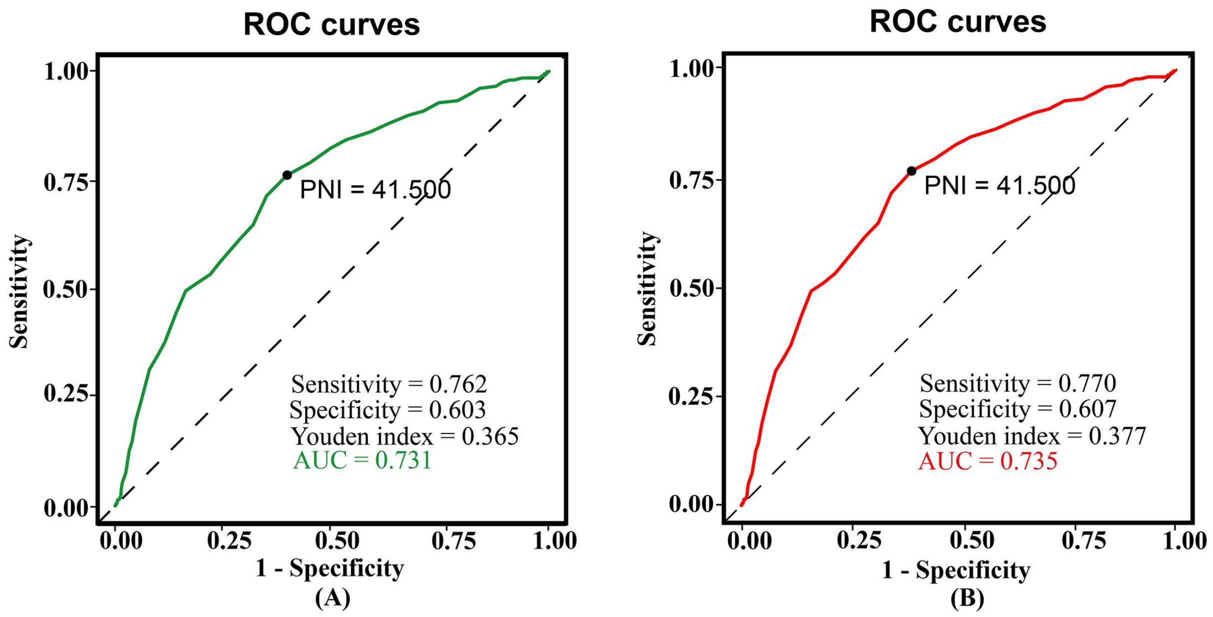

Figure S1 The cut-off value of PNI.

(**A**) about 6-month all-cause mortality; (**B**) about 1-year all-cause mortality.
